# An analysis of COVID-19 vaccination campaign in Ukraine

**DOI:** 10.1093/eurpub/ckad201

**Published:** 2023-12-12

**Authors:** Tetiana Vasylivna Mamontova

**Affiliations:** Physiology Department, Poltava State Medical University, Poltava, Ukraine

## Abstract

**Background:**

The Coronavirus disease (COVID-19) pandemic is still an important problem of concern in Ukraine. The rapid deployment of the vaccination program is a key event for the formation of herd immunity and helps to prevent negative outcomes, overloading the public health system.

**Methods:**

The object of the retrospective-archival study was a depersonalized database of open panels on the management of the COVID-19 situation from the Ministry of Health of Ukraine.

**Results:**

The total number of COVID-19 cases in Ukraine as of 25 December 2022 amounted to 5 314 388 individuals (12.83% of the population), and COVID-19-related deaths reached 110 029 individuals (0.26% of the population). The overall number of COVID-19 vaccinated persons in Ukraine as of 16 January 2022, with one dose was 36 198 per 100 000 persons (36% of the population), and with two doses, it was 34 703 per 100 000 persons (35% of the population). It was shown a decrease in the number of COVID-19 vaccinated persons among men and persons over the age of 70. In the dynamics of COVID-19 vaccination with one and two doses, an increase in the number of persons vaccinated with Pfizer/BioNTech and CoronaVac was noted.

**Conclusions:**

Despite the significant increase in the morbidity and mortality rates of COVID-19, the coverage of vaccination among the population remained insufficient in Ukraine. The effective COVID-19 vaccination strategy should include appropriate management and ensuring the public health system capacity, implementation of information technologies to address logistics issues, and clear communication strategies to maintain public trust.

## Introduction

Reducing the social and medical impact caused by the global increase in COVID-19 morbidity and mortality is one of the most critical priorities for national healthcare systems worldwide. For this purpose, on 24 December 2020, Ukraine developed the COVID-19 vaccination program ‘Roadmap for the Implementation of the Vaccine against Acute Respiratory Disease COVID-19 Caused by the severe acute respiratory syndrome coronavirus 2 (SARS-CoV-2) Coronavirus and Conducting Mass Vaccination in Response to the COVID-19 Pandemic in Ukraine in 2021–22’.^1^ One of the key directions of this program was to ensure adequate and equal access to effective COVID-19 vaccines for the entire population.

Ukraine started the COVID-19 vaccination program a few months after other European countries, on 24 February 2021. The campaign was planned to be conducted in five stages based on prioritizing at-risk groups within the population. Five vaccines are allowed to be used to implement the immunization program against COVID-19: Comirnaty (Pfizer/BioNTech, BNT162b2), CoronaVac (Sinovac Biotech), AstraZeneca (Covishield, SKBio), Moderna (mRNA-1273), and Johnson & Johnson (Janssen Ad26.COV2.S).^2^ However, from the beginning, the campaign encountered numerous factors that seriously hindered its success, including widespread hesitancy regarding vaccination among both the general population and healthcare workers, caused in part by misinformation about vaccine safety and efficacy, undermining public confidence in the authorities, logistical problems in the supply of vaccines.^3^ According to the Ukrainian government’s initial forecasts, it was planned to vaccinate 50% of the population in 2021–22, but with the start of armed conflict, widespread COVID-19 vaccination coverage was not achieved in the country. The full-scale invasion of Ukraine by Russia on 24 February 2022, caused not only the destruction of medical infrastructure but also caused an unprecedented humanitarian crisis throughout the country. During the period of war, there were security issues, destruction of the system, and chains of timely delivery and distribution of vaccines, along with limited mobility for providing immunization services.^4^^,^^5^ Since the beginning of the war, the mass displacement of about 15 million citizens has been registered: 6 million persons were forced to leave the country and 9 million became internally displaced persons.^6^ These population groups are at a high risk of missing or experiencing delays in receiving the necessary vaccine doses, making them highly susceptible to infectious diseases.^7^ However, the experience of the deployment of the COVID-19 vaccination program in Ukraine from the perspective of assessing its effectiveness in preventing morbidity and mortality remains unanalyzed, which strengthened the determination to assess the dynamics of COVID-19 epidemiological characteristics in the country.

The aim of this study was to track the total number of COVID-19 vaccinations with a breakdown into first and second doses, to enhance the understanding of the scale and pace of the vaccination campaign’s deployment in Ukraine.

## Methods

The object of the retrospective-archival study was a depersonalized database of open panels on the management of the COVID-19 situation from the Ministry of Health (MOH) of Ukraine. The epidemiological situation of COVID-19 was assessed from 3 March 2020 (the patient zero case) to 25 December 2022. The deployment of the COVID-19 vaccination campaign was assessed from 24 February 2021 (the start of the campaign) to 16 January 2022.

Epidemiology of the COVID-19 data is presented as weekly new cases of illness, weekly new cases of death, cumulative illness cases and cumulative death cases. The data of the COVID-19 vaccination campaign were determined by weekly new cases of COVID-19 vaccination, divided by the number of vaccine doses [one dose and all doses according to the vaccination protocol (full vaccination)] with distribution by gender, age group and type of vaccine. The age groups of COVID-19 vaccinated persons were divided into intervals as follows: 12–15, 16–19, 20–39, 40–49, 50–59, 60–69, 70–79 and 80+. The state of epidemiology and vaccination against COVID-19 was assessed according to indicators calculated per 100 000 population.

Statistical analysis of data was carried out using the program ‘STATISTICA 10.0’ (StatSoft, Inc., USA). Descriptive analysis, in the form of the sum, median (Me) and interquartile range (IQR), was used for various epidemiological or vaccination indicators, as they have outliers, for some indicators frequency, and percentages were used. Chi-square statistics (χ^2^) were used to test the difference between two independent groups. A *P* value below 0.05 was considered a statistically significant limit.

## Results

### Analysis of the dynamics of morbidity and mortality from COVID-19

**Figure 1:**
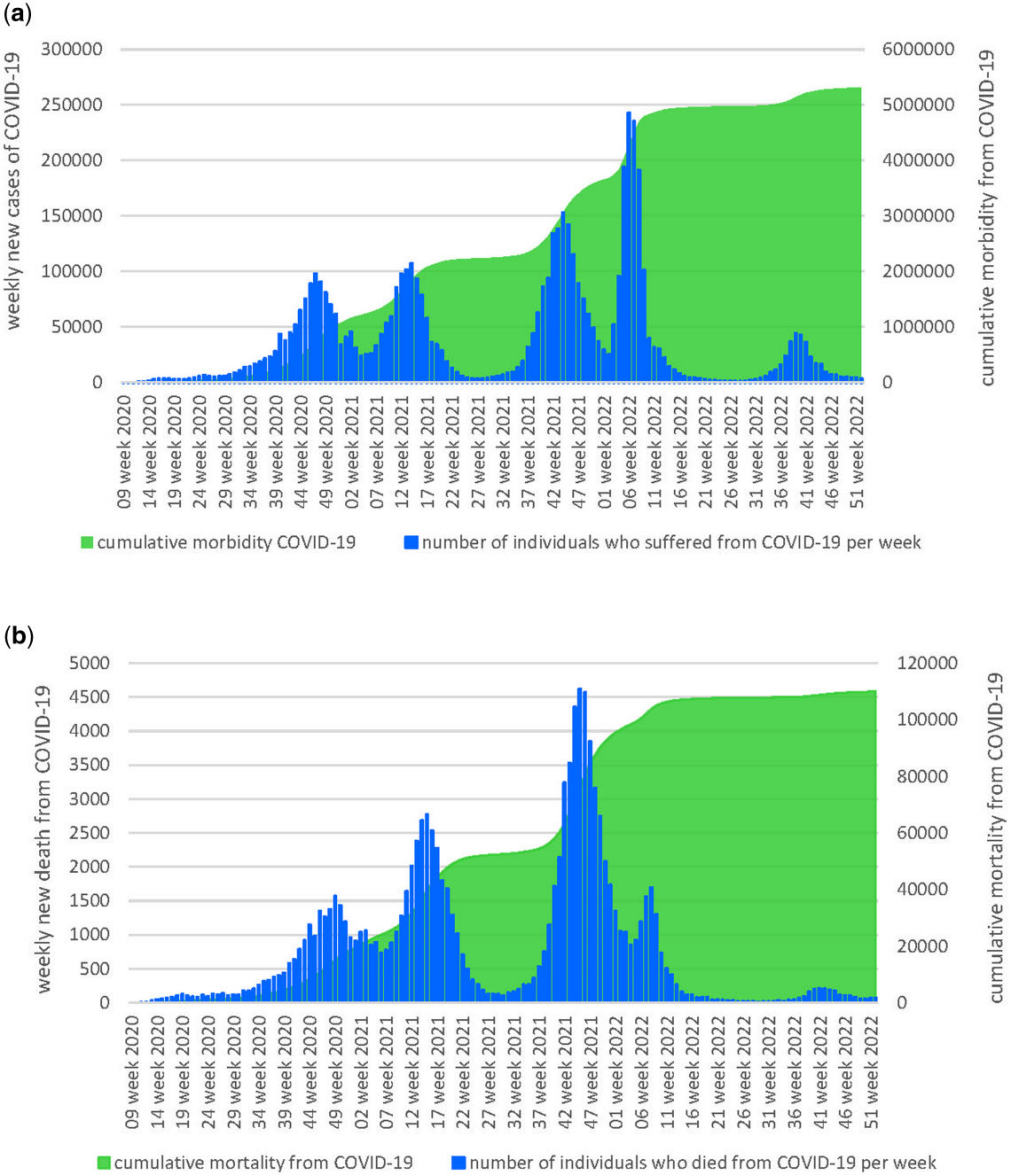
Dynamic of weekly new cases of COVID-19 and cumulative morbidity from COVID-19 (**a**), weekly new deaths from COVID-19 and cumulative mortality from COVID-19 (**b**) in Ukraine in 2020–22 y.y.

In Ukraine, as of 25 December 2022, the cumulative COVID-19 incidence was 5 314 388 persons (12.83% population of Ukraine; Me—16 908; [IQR 43 585–49 059]), the cumulative COVID-19 mortality was 110 029 persons (0.26% population of Ukraine; Me - 263; [IQR 85–1059] (figure 1). At the time of the study, the average COVID-19 incidence in Ukraine was 40.82 per 100 000 persons, the COVID-19 mortality rate was 0.63 per 100 000 persons.

Throughout the course of the pandemic from 2020 to 2022, there have been five peaks of increased weekly COVID-19 morbidity and mortality rates (Supplementary figure S1a and b). The maximum rate of morbidity was observed during the fourth wave of the pandemic—in February 2022, with 24 942 weekly new cases, and the maximum rate of mortality—during the third wave, in November 2021, with 4622 weekly death cases.

From 2020 to 2021, the number of COVID-19 new cases significantly increased by 2.4 times from 1.07 million to 2.55 million persons (*P* < 0.05). In 2022, compared with the previous year, the number of COVID-19 new cases decreased by 0.6 times to 1.69 million persons (*P* > 0.05) (Supplementary table S1).

In the period from 2020 to 2021, the number of COVID-19 deaths significantly increased by 4.1 times, rising from 18 536 persons to 75 572 persons (*P* < 0.05). Subsequently, in 2022, compared with 2021, the COVID-19 mortality rate significantly decreased by 0.21 times to 15 912 persons (*P* < 0.05).

Thus, since the beginning of the COVID-19 pandemic in Ukraine, the epidemiological situation has been marked by a continuous increase in morbidity and mortality among the population, which required the immediate implementation of vaccination measures capable of preventing further negative outcomes. However, the significant reduction in COVID-19 morbidity and mortality rates throughout 2022 in Ukraine suggests an underestimation of the true scale of the pandemic during the wartime period.

### Gender and age indicators of COVID-19

**Figure 2:**
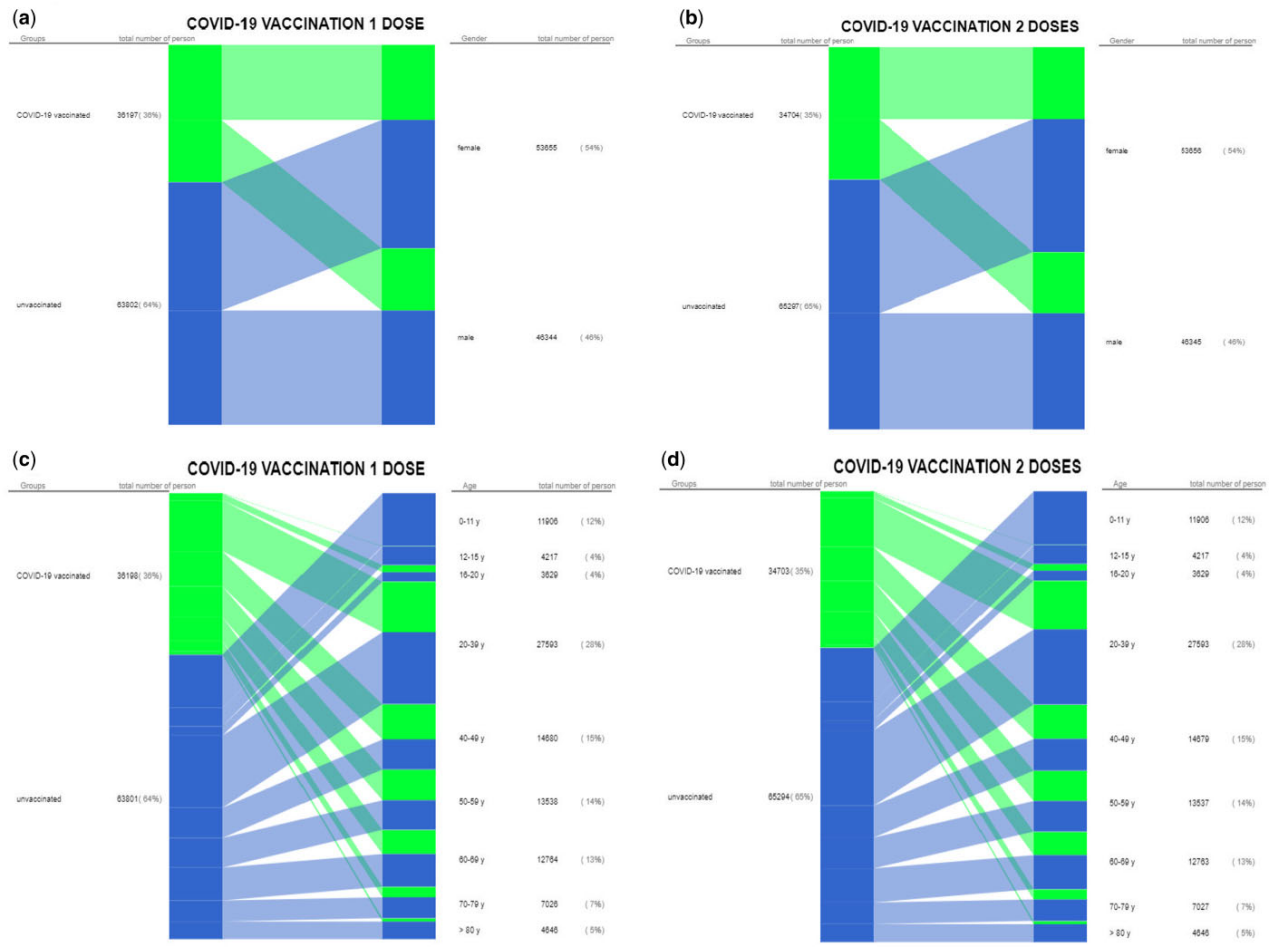
Gender (**a, b**) and age (**c, d**) indicators of the population during COVID-19 vaccination with one and two doses in Ukraine, ×100 000/population

The assessment of gender structure among COVID-19-vaccinated persons revealed heterogeneity between men and women (figure 2a and b). The number of COVID-19-vaccinated women slightly exceeded the number of men while receiving the first and second doses of vaccines (Supplementary table S2).

The first dose of COVID-19 vaccine was received by 16 386 men per 100 000 persons [16% population of Ukraine; Me—78.1; (IQR 11.83–134.6)] and 19 812 women per 100 000 persons [20% population of Ukraine; Me—91.7; (IQR 12.42–167.2)]. The second dose of COVID-19 vaccine was received by 15 905 men per 100 000 persons [16% population of Ukraine; Me—85.35; (IQR 24.55–165.2)] and 18 799 women per 100 000 persons [19% population of Ukraine; Me—103.73; (IQR 25.14–195.5)].

The analysis of the age structure revealed inequality in the distribution of COVID-19 vaccines due to a significant decrease in the number of vaccinated persons among children and adolescents under 19 years and elderly persons over 70 years, regardless of the number of vaccine doses received (figure 2c and d).

COVID-19 vaccines were received by the least number of children aged from 12 to 15 years: 54 per 100 000 persons [0.05% population of Ukraine; Me—0.8; (IQR 0.42–3.6)] vaccinated with the first dose, 46 per 100 000 persons [0.5% population of Ukraine; Me—0.42; (IQR 0.35–4.37)] with the second dose. While elderly persons over 80 years were insufficiently immunized with COVID-19 vaccines: 702 per 100 000 persons [0.7% population of Ukraine; Me—3.1; (IQR 1.19—6.2)] vaccinated with the first dose, 641 per 100 000 persons [0.64% population of Ukraine; Me—3.9; (IQR 1.58–6.57)] with the second dose (Supplementary table S2).

Thus, epidemiological monitoring of demographic indicators indicates an emerging increased risk of severe COVID-19 outcomes among unvaccinated men and elderly individuals. These data confirm the necessity of focusing on a personalized approach, considering the gender and age specifics of the target population, when optimizing the COVID-19 vaccination strategy.

### Assessment of the dynamics and structure of the COVID-19 vaccination campaign

**Figure 3:**
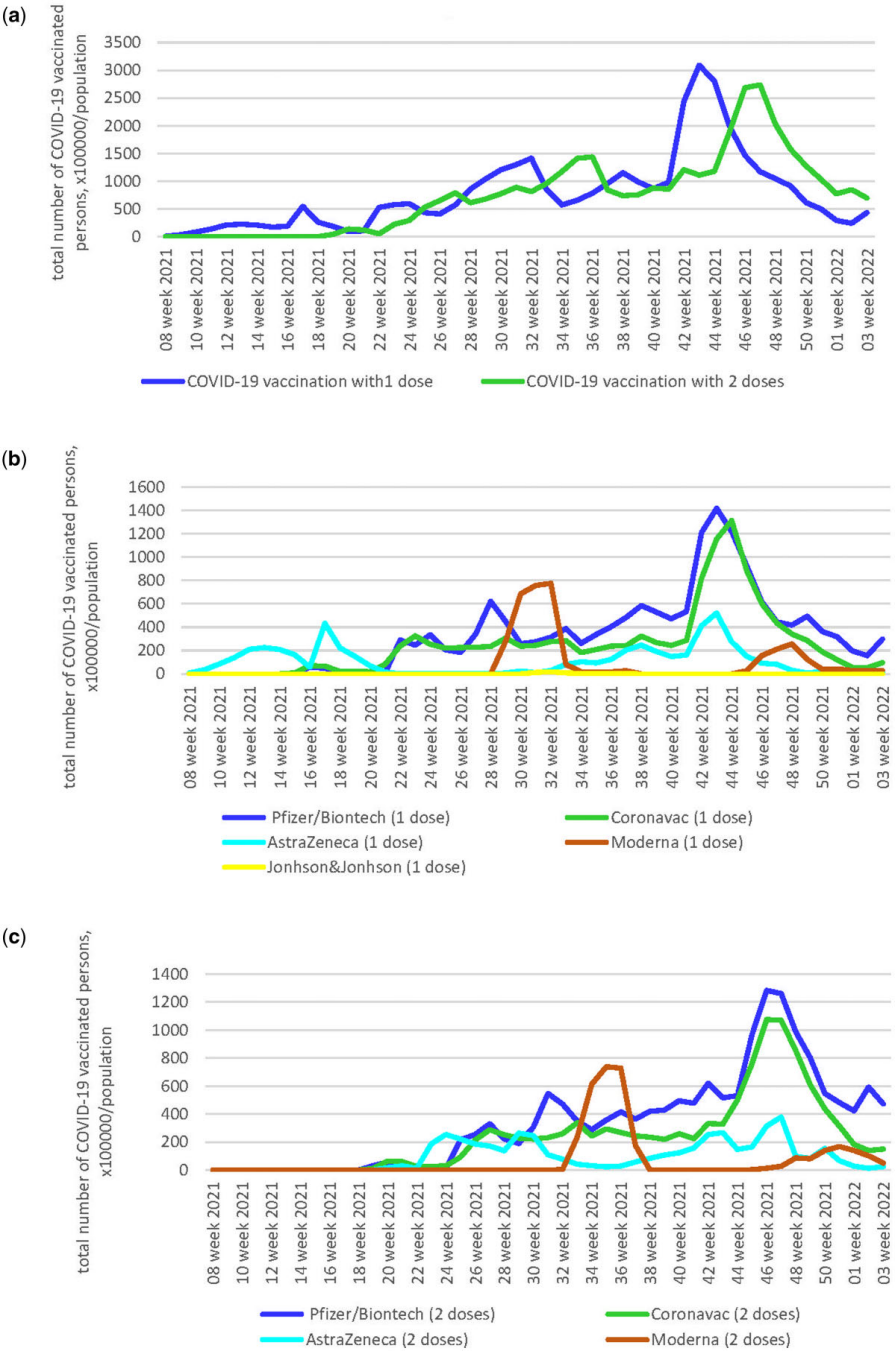
The dynamic of COVID-19 vaccination in Ukraine: common cases with one and two doses (**a**); different types of vaccines with one dose (**b**); different types of vaccines with two doses (**c**), ×100 000/population

Analysis of the COVID-19 vaccination rates from February 2021 to January 2022 showed a gradual increase in the number of persons who received one or two doses of vaccines. In the trends, the highest peak of COVID-19 vaccination occurred in October–November 2021, namely, the most immunized with one dose in the period from 42 to 45 weeks, and two doses in the period from 45 to 48 weeks (figure 3a).

During the period from 24 February 2021 to 16 January 2022, 36 198 per 100 000 persons [36% population of Ukraine; Me—576.3; (IQR 225.4–1043.5)] received one dose of COVID-19 vaccines; 34 703 per 100 000 persons [35% population of Ukraine; Me—755.8; (IQR 43.3–1111.0)] received two doses.

The speed of deploying the COVID-19 vaccination campaign and the dosing of the COVID-19 vaccines is key in achieving target indicators. The structure of the COVID-19 vaccination campaign revealed a temporal and quantitative gap in the population’s demand for different types of vaccines (figure 3b and c). At the beginning of the COVID-19 vaccination campaign in Ukraine, from February to March 2021, for a consecutive period of seven weeks, the single AstraZeneca vaccine (Covishield, SKBio) was used for individuals at critical risk of infection and development of COVID-19 as well as those performing critical functions in response to the COVID-19 pandemic. The next stages of the vaccination campaign were already implemented with five vaccines available to the population, which were obtained through state procurement mechanisms or within the framework of the COVAX international initiative.^8^ The further vaccination process was continued since 13 April 2021, with the CoronaVac vaccine (Sinovac Biotech), since 19 April 2021, with the Comirnaty vaccine (Pfizer/BioNTech, BNT162b2), since 20 July 2021, with the Moderna vaccine (mRNA-1273) and since 4 August 2021, with the Johnson & Johnson vaccine (Janssen Ad26.COV2.S).

A more pronounced increase in demand among the population for Comirnaty (Pfizer/BioNTech, BNT162b2) and CoronaVac (Sinovac Biotech) vaccines compared with other types of vaccines was noted (Supplementary table S3). In comparison, the number of persons vaccinated with Comirnaty (Pfizer/BioNTech, BNT162b2) with the first dose was 15 724 per 100 000 persons [Me—336.4; (IQR 206.7–493.5)], with the second dose—15 771 per 100 000 persons [Me—421.6; (IQR 238.2–539.3)], on the other hand, vaccinated with the CoronaVac vaccine (Sinovac Biotech) by 1.3 times less with the first dose—11 709 per 100 000 persons [Me—237.9; (IQR 108.6–296.9)] and by 1.4 times less with the second dose—11 079 per 100 000 persons [Me—238.9; (IQR 139.5–328.5)].

In general, the administration of the first and second doses of COVID-19 vaccines followed the recommended immunization schedules. It was found that at the beginning of the immunization, there was a tendency to shift toward a delay with an average duration of 4 weeks (from 16 to 19 weeks 2021) between the first and second doses of the Comirnaty vaccine (Pfizer/BioNTech, BNT162b2) (figure 3b and c).

The analysis of COVID-19 vaccination trends in Ukraine during 2021 revealed that despite the steady increase in rates, 36% and 35% of the country’s population received the first and second doses of vaccines, respectively. Among the five types of COVID-19 vaccines, Comirnaty (Pfizer/BioNTech, BNT162b2) and CoronaVac (Sinovac Biotech) had the highest share. The overall assessment of the situation revealed an insufficient level of COVID-19 vaccination to achieve ‘herd immunity’ among the population of Ukraine. The obtained results can have significant implications for determining further directions for improving the strategy of the vaccination campaign.

## Discussion

COVID-19 vaccination has significantly altered the course of the pandemic, saving tens of millions of lives worldwide; however, it has also posed a substantial challenge for governments, public health systems and the population. Understanding the impact of vaccination on the course of the COVID-19 pandemic at a country level is a complex task, given the heterogeneous access of countries to vaccines combined with measurable outcomes, variability within the target cohort, vaccine dosing, development of vaccination infrastructure, changing landscape of circulating SARS-CoV-2 virus strains, implemented national quarantine restrictions, and even more so, especially the impact of armed conflicts. However, an analysis of the vaccination strategy may be important for assessing the effectiveness of the COVID-19 immunization program and identifying ways for its optimization. In this study, trends and the deployment strategy of the vaccination program implemented in Ukraine to protect citizens from the SARS-CoV-2 virus have been investigated.

The start of the COVID-19 vaccination campaign in Ukraine was characterized by a slow start (8th week of 2021), as it began shortly before the increase in the second wave of morbidity and mortality of the pandemic (12th week of 2021) among the country’s population. This situation was caused by numerous disputes between government officials that arose over the purchase of the first doses of vaccines and the clarification of logistical mechanisms for supply, which required storage of the vaccines in ultra-low temperature cold equipment.^9^ The pace of the vaccination campaign significantly accelerated by the 42nd week of 2021, on the eve of the third wave of the pandemic, which was marked by a record-high mortality rate (45th week of 2021—4622 people per week) for the entire period of the COVID-19 pandemic in Ukraine. In general, in 2021, the population of Ukraine was vaccinated against COVID-19 with the first dose of 36% persons and with the second dose of 35% persons. The target indicators declared from the beginning of the COVID-19 vaccination program for 2021, which is 50% of the country’s population, were not achieved. The level of morbidity at the beginning of 2022 reached its record high, amounting to 24 942 new weekly cases among the population of Ukraine during the fourth wave of the pandemic (6th week).

The priority list for COVID-19 vaccine access in Ukraine recommends a predominant focus on professionals (healthcare workers; social workers; persons and workers living in long-term care facilities; military personnel; employees of critical state security structures; teachers and employees in the field of education; persons who are in places of restriction of freedom and employees of these structures) but provided for taking into account age (60 years and older) and health status (adults aged 18–59 with concomitant diseases who are at risk regarding the development of complications and the occurrence of death).^1^^,^^10^ An additional measure to strengthen vaccination efforts was the MOH of Ukraine’s approval on 7 October 2021 of a list of professions for which COVID-19 vaccination is mandatory, encompassing employees in educational institutions and executive authorities (both central and regional).^11^

The demographic analysis of COVID-19 vaccinations reveals an imbalance by age and gender categories, showing a decline in the proportion of men and older individuals receiving both first and second doses. According to the MOH of Ukraine data, during the 10th week of 2020–21st week of 2021, women prevailed in the number of confirmed COVID-19 cases (59.5% of persons), and the elderly over 70 years—in the number of deaths (52.5% of persons).^1^ The results showed that women were more willing than men to be vaccinated against COVID-19, despite high levels of distrust in the safety and efficacy of vaccines among women at the start of the campaign.^12^ The national vaccination program considered the elevated risks for specific age groups and suggested vaccination for those over 80 years during the second stage and for those aged 65–79 years old during the third stage. The vaccination data for the elderly reveals a concerning trend: In 2021, one and two vaccine doses of vaccine received by only 2.31% and 2.24% of persons aged between 70 and 79 years, respectively, and a mere 0.7% and 0.64% of those above 80 years, respectively. These data show that the vaccination coverage target for persons aged 60 years or more (9 978 194 persons, 24.1% population of Ukraine)^1^ was not achieved. The low level of COVID-19 vaccination among the elderly serves as a clear signal to strengthen the targeted monitoring and information on the vaccination of this age group.

The President and Government of Ukraine made significant efforts to promote the vaccination program and obtain COVID-19 vaccines. Already in March 2021, the campaign was forced to overcome a high level of distrust among the population, which, according to the United Nations Development Program (UNDP) and the United Nations Children’s Fund (UNICEF), was significantly increased by the uncontrolled infodemic about COVID-19 in Ukrainian online mass media, blogs, forums, social networks and messaging platforms.^13^^,^^14^ To overcome these problems, directions for responding to the situation have been formed through the involvement of influencers and leaders to encourage public confidence in vaccines, informing through the media, and encouraging at the local level. On 19 December 2021, the Presidential program ‘eSupport’ was launched to financially reward people for getting vaccinated against the coronavirus.^15^

In early 2021, the Ukrainian Government signed agreements to purchase COVID-19 vaccines from several manufacturers, including 1 913 000 doses of CoronaVac vaccine (Sinovac Biotech), 20 001 150 doses of Comirnaty vaccine (Pfizer/BioNTech, BNT162b2) and 12 000 000 doses of AstraZeneca (Covishield, SKBio). Ukraine’s partnership with COVAX, a global vaccine-sharing scheme supported by WHO, the Coalition for Epidemic Preparedness Innovations (CEPI), Gavi and UNICEF, has been a significant contribution to the distribution of vaccines. Through this program, about 8 million doses of vaccines from various manufacturers [Comirnaty (Pfizer/BioNTech), Moderna (mRNA-1273), AstraZeneca (Covishield, SKBio), CoronaVac (Sinovac Biotech), Johnson & Johnson (Janssen Ad26. S)] were delivered to the country free of charge at different times throughout 2021.^8^^,^^16^^,^^17^ However, due to the supply of vaccines under the COVAX mechanism for 183 countries, including Ukraine, delays in vaccines and the formation of a longer interval between the first and second doses could be unavoidable in several countries. In Ukraine, immunization of the Comirnaty (Pfizer/BioNTech, BNT162b2) was delayed by an average of 4 weeks instead of the recommended 21 days.^18^

Analysis of the structure of different types of vaccines against COVID-19 during 2021 showed a significant advantage of Comirnaty (Pfizer/BioNTech) and CoronaVac (Sinovac Biotech) among the vaccinated population of Ukraine. Thus, the first dose of the Comirnaty (Pfizer/BioNTech) vaccine was received by 15.7% of the population, CoronaVac (Sinovac Biotech) – 11.7% of the population, AstraZeneca (Covishield, SKBio) – 5.1% of the population, Moderna (mRNA-1273) – 3.6% of the population, Johnson & Johnson (Janssen Ad26.COV2.S) – 0.04% of the population. The second dose of the Comirnaty (Pfizer/BioNTech) vaccine was received by 15.8% of the population, CoronaVac (Sinovac Biotech) – 11.1% of the population, AstraZeneca (Covishield, SKBio) – 4.5% of the population, Moderna (mRNA-1273) – 3.2% of the population. The prioritization of these two vaccine types is probably due to the high efficacy rates of the Pfizer/BioNTech and CoronaVac vaccines during Phase 3 clinical trials in preventing severe disease and death (80–100% and 90–99% of cases; 88–100% and 86% of cases, respectively) and reducing its impact on public health.^19^

A potential limitation of our study may arise in the completeness of the vaccination dataset for the year 2022. The dataset on the epidemiological and vaccination of COVID-19 relies on the information provided by the MOH of Ukraine and other official sources. It relies on the country making the latest figures on administered doses available in a timely manner. Also, Ukrainian organizations provide daily or weekly updates on the different web platforms that complicate the interpretation of reported data. In Ukraine, access to COVID-19 vaccination data was closed with the beginning of the war on 24 February 2022. This affected the accuracy and comprehensiveness of our analysis in assessing the speed of the vaccination rollout after the onset of the war. It’s important to acknowledge this limitation and interpret the results within this context.^20^

Analyzing the data, it can be concluded that since the beginning of the deployment of the COVID-19 vaccination campaign, Ukraine has been faced with solving several problems related to the procurement, receipt of vaccines and the establishment of communication within the country. Notwithstanding having signed contracts to supply COVID-19 vaccines and participating in the COVAX program, the majority of the doses were not received until April 2021,^8^ which did not allow the country to fully realize its potential at the desired pace of the campaign. At the beginning of the campaign, communication strategies were irregular, with disagreements between government officials and the infodemic increasing public concerns about the safety and effectiveness of COVID-19 vaccines. However, changes in the narrative in the political space helped to effectively overcome the problems. Despite these challenges, efforts were made to address the issues and improve the vaccination strategy, as evident from various initiatives and programs introduced to enhance public trust, accessibility, and coverage of vaccination.

Thus, the effective rollout of the national COVID-19 vaccination program in Ukraine should focus on finding ways to ensure proper funding and management, reliable public healthcare system support, early investment in vaccine production, implementing information technologies for logistics solutions, and clear and consistent communication strategies to sustain public confidence. Addressing these aspects will contribute to a more successful and efficient vaccination campaign, ultimately leading to increased vaccination coverage and improved control of the pandemic.

## Supplementary Material

ckad201_Supplementary_Data

## Data Availability

The data underlying this article were derived from a source in the open public domain of the dashboard of COVID-19 of the Ministry of Public Health of Ukraine. Key points In Ukraine, morbidity and mortality rates related to COVID-19 remain high due to insufficient rates of COVID-19 vaccination with both one and two doses in 2021. There has been a decline in the number of COVID-19 vaccinated persons among men, children and adolescents under the age of 19, and elderly persons over 70 years of age were noted, regardless of the number of vaccine doses received in 2021. The vaccination campaign in Ukraine between February 2021 and January 2022 encountered challenges in procuring and distributing vaccines, as well as establishing communication to strengthen public trust in vaccines.
