# Supplementary material for: An analysis of COVID-19 vaccination campaign in Ukraine
Source: Eur J Public Health. 2023 Dec 12;34(1):156–62. doi: 10.1093/eurpub/ckad201 (PMC10843935; doi:10.1093/eurpub/ckad201)
Supplement: ckad201_Supplementary_Data [file ckad201_supplementary_data.pdf]

Supplementary material

Figure S1. The dynamic of morbidity (1a) and mortality (1b) during the COVID-19 pandemic in Ukraine

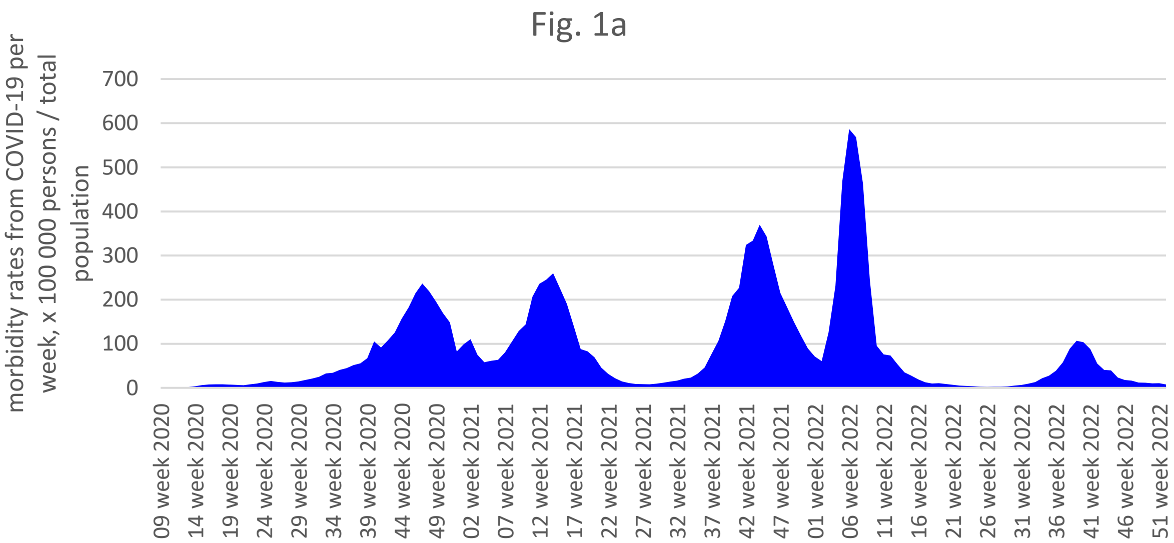

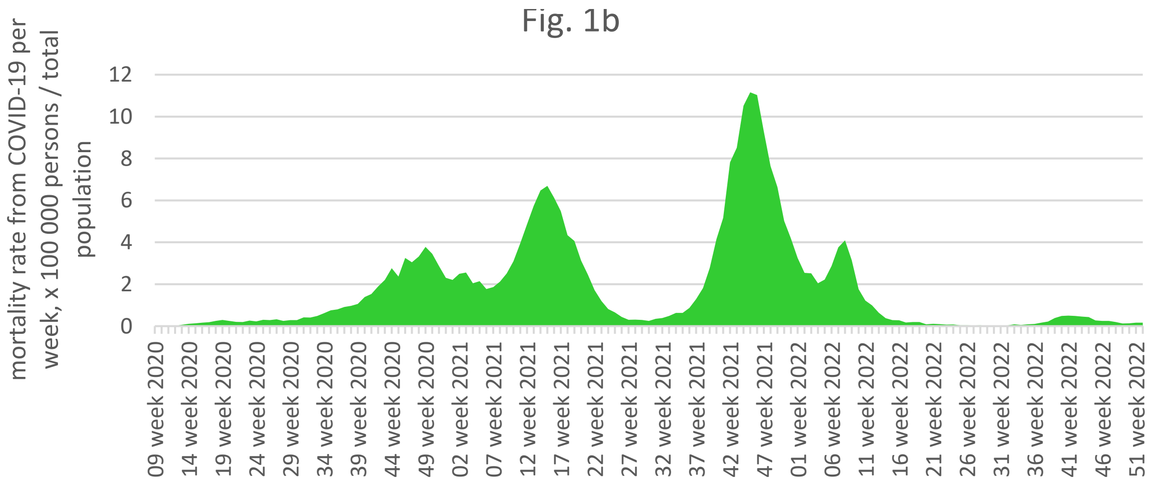

Table S1. Total morbidity and mortality (cases per year) from COVID-19 in 2020- 2022 y.y. in Ukraine

Indicators	Year of the COVID-19 pandemic									p
	2020			2021			2022			
	Sum	Median	IQR Lower - Upper	Sum	Median	IQR Lower - Upper	Sum	Median	IQR Lower - Upper	
Number of individuals who suffered from COVID-19 per week	1068800	8050.0	3233,0 -40705	2552594	36973	13045 - 85607	1692996	9350	3741.5 -30880.5	$p_1<0.05$ $p_2>0.05$
Morbidity rates from COVID-19 per week, x 100 000 persons / total population	2580	19.44	7.81 -98.3	6163	89.27	31.5 -206.69	4088	22.57	9.03 -74.56	$p_1<0.05$ $p_2>0.05$
Number of individuals who died from COVID-19 per week	18536	0.37	90 – 709.5	75572	1040	359 -2273	15912	86	38 -236	$p_1<0.05$ $p_2<0.05$
Mortality rate from COVID-19 per week	45	0.37	0.22 -1.71	182	2.51	0.87 -5.49	38	0.21	0.09 -0.57	$p_1<0.05$ $p_2<0.05$

Note: *p_1 – indicators of the 2020 year in comparison with indicators of the 2021 year of the COVID-19 pandemic*
p_2 - indicators of the 2021 year in comparison with indicators of the 2022 year of the COVID-19 pandemic

Table S2. COVID-19 vaccination cases count with one or two doses by gender and age groups, x100 000 persons/population

Indicators	COVID-19 vaccination with 1 dose				COVID-19 vaccination with 2 doses			
	Sum	% total population	Median	IQR Lower - Upper	Sum	% total population	Median	IQR Lower - Upper
Gender:								
Male	16385	16	78.1	11.83- 134.6	15905	16	85.35	24.55 – 165.2
Female	19812	20	91.7	12.42- 167.2	18799	19	103.73	25.14 – 195.5
Age:								
12-15 y.	54	0.05	0.8	0.42- 3.6	46	0.5	0.42	0.35- 4.37
16-19 y	1561	1.56	4.2	0.95- 9.8	1436	1.44	4.55	1.23- 10.92
20-39 y	11388	11.39	49.0	7.78- 89.9	10828	10.83	54.16	15.86- 119.86
40-49 y	7898	7.9	36.4	5.32- 64.6	7606	7.61	41.06	11.72- 83.2
50-59 y	6888	6.89	32.3	4.14- 60.0	6721	6.72	39.18	9.3- 72.99
60-69 y	5393	5.39	27.0	4.14- 49.6	5182	5.18	36.5	8.44- 52.19
70-79 y	2314	2.31	10.1	2.38- 21.6	2244	2.24	14.2	3.43- 23.79
> 80 y	702	0.7	3.1	1.19- 6.2	641	0.64	3.9	1.58- 6.57

Table S3. COVID-19 vaccination cases count with one or two doses by type of vaccine, x100 000 persons/population

Indicators	COVID-19 vaccination with 1 dose			COVID-19 vaccination with 2 doses		
	Sum	Median	IQR Lower - Upper	Sum	Median	IQR Lower - Upper
Comirnaty (Pfizer/BioNTech, BNT162b2)	15 724	336.4	206.7-493.5	15 771	421.6	238.2-539.3
CoronaVac (Sinovac Biotech)	11 709	237.9	108.6-296.9	11 079	238.9	139.5-328.5
AstraZeneca (Covishield, SKBio)	5 108	81.6	8.85-164.5	4 556	84.4	24.0-171.7
Moderna (mRNA-1273)	3 606	43.9	26.5-256.7	3297	86.3	3.1-175.5
Jonhson&Jonhson (Janssen Ad26.COV2.S)	50	1.7	0.3-6.3			
